# Investigating hospital heterogeneity with a multi-state frailty model: application to nosocomial pneumonia disease in intensive care units

**DOI:** 10.1186/1471-2288-12-79

**Published:** 2012-06-15

**Authors:** Benoit Liquet, Jean-François Timsit, Virginie Rondeau

**Affiliations:** 1, Univ. Bordeaux, ISPED, centre INSERM U-897-Epidémiologie-Biostatistique, Bordeaux, F-33000, FRANCE; 2, INSERM, ISPED, centre INSERM U-897-Epidémiologie-Biostatistique, Bordeaux, F-33000, FRANCE; 3, University-Grenoble 1, institut Albert Bonniot U823- team 11: outcome of mechanically ventilated patients and respiratory cancers, Grenoble, 38041, France; 4, University-Grenoble 1, teaching hospital Albert Michallon, Medical intensive care unit, Grenoble, 38043, France

## Abstract

**Background:**

Multistate models have become increasingly useful to study the evolution of a patient’s state over time in intensive care units ICU (e.g. admission, infections, alive discharge or death in ICU). In addition, in critically-ill patients, data come from different ICUs, and because observations are clustered into groups (or units), the observed outcomes cannot be considered as independent. Thus a flexible multi-state model with random effects is needed to obtain valid outcome estimates.

**Methods:**

We show how a simple multi-state frailty model can be used to study semi-competing risks while fully taking into account the clustering (in ICU) of the data and the longitudinal aspects of the data, including left truncation and right censoring. We suggest the use of independent frailty models or joint frailty models for the analysis of transition intensities. Two distinct models which differ in the definition of time *t* in the transition functions have been studied: semi-Markov models where the transitions depend on the waiting times and nonhomogenous Markov models where the transitions depend on the time since inclusion in the study. The parameters in the proposed multi-state model may conveniently be computed using a semi-parametric or parametric approach with an existing R package FrailtyPack for frailty models. The likelihood cross-validation criterion is proposed to guide the choice of a better fitting model.

**Results:**

We illustrate the use of our approach though the analysis of nosocomial infections (ventilator-associated pneumonia infections: VAP) in ICU, with “alive discharge” and “death” in ICU as other endpoints. We show that the analysis of dependent survival data using a multi-state model without frailty terms may underestimate the variance of regression coefficients specific to each group, leading to incorrect inferences. Some factors are wrongly significantly associated based on the model without frailty terms. This result is confirmed by a short simulation study. We also present individual predictions of VAP underlining the usefulness of dynamic prognostic tools that can take into account the clustering of observations.

**Conclusions:**

The use of multistate frailty models allows the analysis of very complex data. Such models could help improve the estimation of the impact of proposed prognostic features on each transition in a multi-centre study. We suggest a method and software that give accurate estimates and enables inference for any parameter or predictive quantity of interest.

## Background

Multistate models have become increasingly useful to understand complicated biological processes and to evaluate the relations between different types of events. These methods have been developed to study simultaneously several competing causes of failure (e.g. competing risks of death) or to study the evolution of a patient’s state over time (e.g. admission in intensive care units (ICU), infections, alive discharge or death in ICU) and the focus is in the process of going from one state to another.

Furthermore, many studies include clustering of survival times. For instance, in critically ill patients, data come from different ICUs and because observations are clustered into groups (or units), the observed outcomes cannot be considered as independent. Thus a flexible multi-state model with random effects is needed to obtain valid outcome estimates. Ignoring the existence of clustering may underestimate the variance of regression coefficients specific to each group, leading to incorrect inferences.

Ripatti et al. [1] proposed a three-state frailty model to model age at onset of dementia and death in Swedish twins. The intra-pairs correlations and the other parameters were estimated using hierarchical bayesian model formulation and Gibbs sampling, both of which can be time-consuming. Katsahian et al. [2,3] extended Fine and Gray’s [4] model to the case of clustered data, by including random effects in the subdistribution hazards. They first used the residual maximum likelihood then the penalized partial log-likelihood to estimate the parameters. However, the estimation approach does not directly yield estimators of the transition intensities, which often have a meaningful interpretation in epidemiological studies. Most of the time, the baseline intensity estimate is based on Breslow’s estimate leading to a piecewise-constant baseline hazard function or unspecified baseline hazard function.

In this paper, we show how a simple multi-state frailty model can be used to study semi-competing risks [5] while fully taking into account the clustering (in ICU) of the data and the longitudinal aspects of the data, including left truncation and right censoring. We suggest the use of independent frailty models or joint frailty models for the analysis of transition intensities. This approach is of interest for several reasons. First, it allows to deal with heterogeneity between ICUs. We do this by including cluster-specific random effects or frailties in the multi-state model. Frailties represent the unmeasured covariates at the cluster level, which may affect the rate of occurrence of each of the events. Moreover, this approach allows us to evaluate different prognostic effects of covariates on each transition probability. Another interesting and perhaps underrated advantage of multi-state models is the possibility to use them to predict clinical prognosis whereby a patient will be in a given health state at time *u* given a particular history at time *t*. This work extends previous research by dealing with clustered competing risks and by giving smoothed estimates of the transition rates. In addition the joint approach allows the analysis of two processes that evolve with time leading to more accurate estimates.

Two distinct approaches are often used in multi-state models. They differ in the definition of time *t* in the transition functions. In the first approach, the transition probability between two states depends only on the waiting times, the clock is reset to zero every time a patient enters a new state and a semi-Markov model is used. In the second approach, the transition depends only on the time since inclusion in the study, and nonhomogenous Markov models are used. The proposed approach can deal with both situations and is illustrated in this article. The choice between one the two approaches depends on the clinical knowledge of the events of interest. If it is expected that the transition probability (for instance toward death) will not change as a function of the time since randomization or inclusion, the analysis can be based solely on the semi-Markov model and it can thus be studied how the transition probability evolves after an event has taken place (for instance after nosocomial infections). However, if it is difficult to choose clinically between the semi-Markov or the nonhomogeneous Markov approach, one can use statistical criteria [6].

As discussed in the section on the *estimation procedure* method, an important advantage of our proposed approach is that the parameters in the multi-state model may conveniently be computed using a semi-parametric or parametric approach with an existing R package FrailtyPack for frailty models.

The paper is organized as follows. First, the ICU data is briefly presented. The next section describes the statistical multistate frailty model for clustered data with estimation procedure. Then, the model is applied to the analysis of nosocomial infections (ventilator-associated pneumonia infections) in ICUs, with “alive discharge” and “death” in ICU as other endpoints. Results from a simulation study are reported. Finally, a concluding discussion is presented.

## Methods

### Motivating example

#### Data Source

We conducted a prospective observational study using data from the multi-center **Outcomerea** database between November 1996 to April 2007. The database contains data from 16 French ICUs, among which data on admission features and diagnosis, daily disease severity, iatrogenic events, nosocomial infections, and vital status. Every year, the data of a subsample of at least 50 patients per ICU were entered in the database; patients had to be older than 16 years and to have stayed in ICU for more than 24 hours. To define this random subsample, each participating ICU selected either consecutive admissions to specific ICU beds throughout the year or consecutive admissions to all ICU beds over a single month.

#### Data collection

Database quality measures were taken such as the continuous training of investigators in each ICU or regular data quality checks (see [7]). A one day coding course was organized annually with the study investigators and research monitors. In all ICUs, as previously reported [8,9], VAP was suspected based on the development of persistent pulmonary infiltrates on chest radiographs combined with purulent tracheal secretions, and/or body temperature ≥ 38.5°C or ≤ 36.5°C, and/or peripheral blood leukocyte count ≥ 10 × 10^9^ / *L*(Giga/liter) or ≤ 4 × 10^9^ / *L*. The definite diagnosis of VAP required a positive culture result from a protected specimen brush (≥ 10^3^*cfu*/*ml*), plugged telescopic catheter specimen (≥ 10^3^*cfu*/*ml*), BAL fluid specimen (≥ 10^4^*cfu*/*ml*), or quantitative endotracheal aspirate (≥ 10^5^*cfu*/*ml*). Investigators systematically performed bacteriological sampling before changing antimicrobials.

#### Study population

We considered death in ICU and discharge to be absorbing state and VAP as a non absorbing state. Patients were included in the study if they had stayed in the ICU for at least 48 hours and had received mechanical ventilation (MV) within 48 hours after ICU admission. We obtained 2871 patients, corresponding to 37395 ICU days. The median MV duration was 7 days with inter quartile range (IQR = [4-13]).

### The multi-state approach and estimation

#### Multi-state model

**Figure 1:**
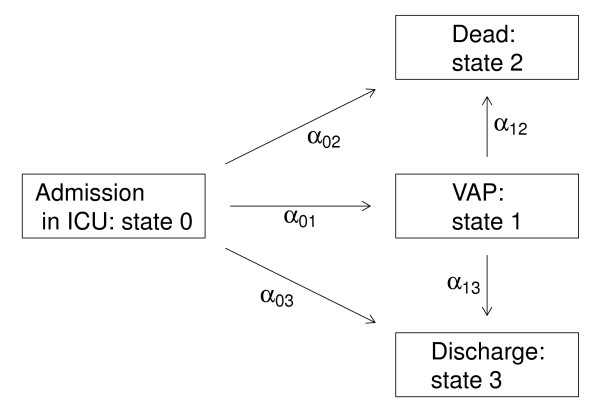
The “disability Model”.

We consider the multi-state model represented in Figure 1 corresponding to our motivating example. This discrete stochastic process (*X*(*t*))_*t* ≥ 0_ with state space {0,1,2,3} called *disability model* is often used to describe the occurrence of nosocomial infections in ICU [10]. Here we focus on the occurrence of VAP modelled via the transition 0 (admission in ICU without VAP) into state 1 (VAP infections). Discharge alive and death in ICU are modelled via transitions into states 3 and 2, respectively. A transition will be simply denoted *hk*. The set of transitions for the *disability model* will be denoted E ( E:={01,02,03,12,13}). The distribution of this multi-state process is characterized by the transition intensities:

(1) $$\alpha_{\mathrm{hk}}(t;F_{t^{-}})=\underset{\mathrm{\Delta t}\to 0^{+}}{\text{lim}}\frac{P_{\mathrm{hk}}(t,t+\mathrm{\Delta t};F_{t^{-}})}{\mathrm{\Delta t}},\;\;\mathrm{hk}\in E,$$

where $P_{\mathrm{hk}}(t,t+\mathrm{\Delta t})=P(X(t+\mathrm{\Delta t})=k|X(t)=h;F_{t^{-}})$ are the transition probabilities. The filtration $F_{t^{-}}$ stands for the history of the process just before time *t* (*t*^−^).

From the definition of the transition intensities, we focus on two kinds of models. First, we consider the *non-homogeneous Markov model* implying that the transition intensities depend only on the current time *t*. In our application, the time *t* represent time since entry in ICU (and for all patients *X*(0) = 0). So for instance, when death occurs after VAP infections, *t* represents time since ICU entry, and not time since infections. We obtain:

(2) $$\alpha_{1k}(t;F_{t^{-}})\;=\;\underset{\mathrm{\Delta t}\to 0^{+}}{\text{lim}}\frac{P_{1k}(t,t+\mathrm{\Delta t})}{\mathrm{\Delta t}}\;=\;\alpha_{1k}(t),\;k=2,3,$$

depends on *t* but not on the entry time into state 1 (noted *T*_1_ in the sequel). Second, we define the *semi-Markov model* implying that the transitions intensities depend only on the time spent in the current state. In particular, we get in our application:

(3) $$\;\alpha_{1k}(t;F_{t^{-}})\;=\underset{\mathrm{\Delta t}\to 0^{+}}{\text{lim}}\frac{P_{1k}(t,t+\mathrm{\Delta t})}{\mathrm{\Delta t}}\;=\;\alpha_{1k}(t-T_{1}),k=2,3.$$

#### Intensity functions with a shared frailty term

Take the case of a study with *G*-independent intensive care units (*i* = 1,…,*G*). Let $Z_{\mathrm{hk}}^{\mathrm{ij}}$ be a vector of covariates (specific to the transition *hk*) for subject *j* (*j* = 1,…,*n*_*i*_) from group *i*. In order to take into account the heterogeneity of the population due to the fact data come from different ICUs, we simply model the transition intensities by a proportional intensities model with frailty. For any hk∈E, the transition intensity for the *j*th patient in the *i*th ICU with random ICU effects $u_{\mathrm{hk}}^{i}$ given a vector of covariates $Z_{\mathrm{hk}}^{\mathrm{ij}}$ is defined by

(4) $$\alpha_{\mathrm{hk}}^{\mathrm{ij}}(t|u_{\mathrm{hk}}^{i},Z_{\mathrm{hk}}^{\mathrm{ij}})=\alpha_{0,\mathrm{hk}}^{\theta_{\mathrm{hk}}}(t)u_{\mathrm{hk}}^{i}\exp(\beta_{\mathrm{hk}}^{T}Z_{\mathrm{hk}}^{\mathrm{ij}})\;\mathrm{hk}\in E,$$

where $\left(\alpha_{0,\mathrm{hk}}^{\theta_{\mathrm{hk}}}(t)\right)$ is the baseline transition intensity specific to the transition *hk* (with specific parameter *θ*_*hk*_ for parametric model) and the random ICU effects $u_{\mathrm{hk}}^{i}$ are also specific to the transition *hk*, independent and follow a Gamma distribution ( $u_{\mathrm{hk}}^{i}∼\Gamma \left(\frac{1}{\gamma_{\mathrm{hk}}},\frac{1}{\gamma_{\mathrm{hk}}}\right)$, $E[u_{\mathrm{hk}}^{i}]=1$, $\mathrm{Var}[u_{\mathrm{hk}}^{i}]=\gamma_{\mathrm{hk}}$). The variance *γ*_*hk*_ of the $u_{\mathrm{hk}}^{i}$ represents the heterogeneity between ICU of the overall underlying baseline risk for the transition *hk*.

##### Remark

this definition is correct for the *non-homogeneous Markov model*. We get a similar definition for the *semi-Markov model* by replacing the current time *t* in the equation (4) by the time spent in the current state *t* − *T*_1_(for the transition 12 and 13).

#### Intensity functions with a joint frailty term

In the model (4) we assume that the different times to transitions are independent. In some cases this assumption may be violated, for instance in our motivating example, the transition times to death with the VAP and the transition times to discharge with VAP may be dependent. This dependency should be accounted for in the joint modelling of these two survival endpoints. There can be many reasons to use joint models of two survival endpoints, including giving a general description of the data, correcting for bias in survival analysis due to dependent dropout or censoring, and improving efficiency of survival analysis due to the use of auxiliary information [11].

In this work we will thus also consider some transitions jointly, in a joint frailty model setting as follows:

(5) $$\begin{array}{lcrr} \left\{\begin{array}{l} \alpha_{\mathrm{hk}}^{\mathrm{ij}}(t|\omega_{j},Z_{\mathrm{hk}}^{\mathrm{ij}})=\alpha_{0,\mathrm{hk}}^{\theta_{\mathrm{hk}}}(t)\omega_{j}\;\text{exp}(\beta_{\mathrm{hk}}^{T}Z_{\mathrm{hk}}^{\mathrm{ij}})\\ \alpha_{{\mathrm{hk}}^{\prime }}^{\mathrm{ij}}(t|\omega_{j},Z_{{\mathrm{hk}}^{\prime }}^{\mathrm{ij}})=\alpha_{0,hk^{\prime }}^{\theta_{{\mathrm{hk}}^{\prime }}}(t)\omega_{j}^{\zeta }\;\text{exp}(\beta_{{\mathrm{hk}}^{\prime }}^{T}Z_{{\mathrm{hk}}^{\prime }}^{\mathrm{ij}}) \end{array}\right) & & & \end{array}$$

The random effects *ω*_*j*_ (frailties) are assumed independent. Mainly for reasons of mathematical convenience, the frailty terms are often assumed to follow a gamma distribution. The gamma frailty density is adopted here with unit mean and variance *η*. This choice and other possibilities such as log-normal, positive stable distributions are discussed in several papers [12,13]. The common frailty parameter *ω*_*j*_ will take into account the heterogeneity of the data, associated with unobserved covariates.

In the traditional model, the assumption is that *ζ* = 0 in (5), that is *α*_*hk*_(*t*) does not depend on $\alpha_{{\mathrm{hk}}^{\prime }}(t)$, and thus the two intensity functions *α*_*hk*_(*t*) and $\alpha_{{\mathrm{hk}}^{\prime }}(t)$ are not associated, conditional on covariates.When *ζ* = 1, the effect of the frailty is identical for the two transition times. When *ζ* > 0, the two transition times are positively associated; higher frailty will result for instance in a higher risk of discharge and a higher risk of death; while *ζ* < 0 implies a negative association. This means that unobserved individual factors produce higher transition rates to death and smaller transition rates to discharge, or inversely. We can think that for sicker patients, the mortality will be high but with a low discharge rate, conversely for healthy patients, the discharge rates will be high with a low death rate. The interpretation of the value of *ζ* only makes sense in case of heterogeneity, i.e. when the variance of the random effects is significantly different from zero. However, in this model we assume that there is no intra-cluster correlation anymore after having taken into account prognostic factors and after adjusting for a subject specific random effect term.

#### Estimation procedure

First, in our study, we consider that the process (*X*(*t*))_*t* ≥ 0_ is observed in continuous time, i.e., we know at each time *t* the state of the process for each subjects. Secondly, for the model with shared frailties terms, we specify that each transition intensity has its own set of parameters. In other words, the parameter *θ*_*hk*_, *β*_*hk*_ and *γ*_*hk*_ are specific to the transition *hk*. Such an assumption is common when dealing with multi-state models and [14] has shown that this assumption allows us to consider the problem of estimating (parametrically or semi-parametrically) the transition intensities separately (that is transition by transition). Thus, each transition intensity can be evaluated by estimation methods used in survival analysis in the presence of right-censored data only (for instance when *semi-Markov models* are used) or in the presence of right-censored and left-truncated data (for instance when *non-homogeneous Markov models* are used, the transition intensities 12 and 13 are evaluated using delayed entry).

In this paper, we use two different approaches. First, we use a parametric model for the multi-state model specifying each baseline transition intensity by a Weibull distribution ( $\left(\alpha_{0,\mathrm{hk}}^{\theta_{\mathrm{hk}}}\left(t\right)=\left(a_{\mathrm{hk}}/b_{\mathrm{hk}}\right){\left(t/b_{\mathrm{hk}}\right)}^{\left(a_{\mathrm{hk}-1}\right)}\right)$; *θ*_*hk*_ = (*a*_*hk*_, *b*_*hk*_)). For each transition, we estimate the different parameter (*ζ*_*hk*_ = (*θ*_*hk*_, *β*_*hk*_, *γ*_*hk*_)) by maximizing the full marginal log-likelihood [15]. In this case, the full log likelihood for right-censored data and left-truncated data takes a simple form with an analytical solution for the integrals [15]. In practice, we use the FrailtyPack R package [16] to estimate all parameters in the different multi-state model performed [17]. Secondly, we consider a semi-parametric estimation by introducing a semi-parametric penalized likelihood approach to estimate the different parameters *β*_*hk*_, *γ*_*hk*_ and the baseline intensity function *α*_0,*hk*_(*t*). This approach is more flexible than a too rigid parametric approach to estimate a smooth baseline intensity function. To obtain a smooth baseline intensity function, we penalize the likelihood by the squared norm of the second derivative of the intensity function. The penalized log-likelihood for the transition *hk* is thus defined as

(6) $$\begin{array}{ccc} \;\mathrm{pl}(\beta_{\mathrm{hk}},\gamma_{\mathrm{hk}},\alpha_{0,\mathrm{hk}}(\cdot )) & \;= & l(\beta_{\mathrm{hk}},\gamma_{\mathrm{hk}},\alpha_{0,\mathrm{hk}}(\cdot )\\ & & -\kappa_{\mathrm{hk}}\int_{0}^{\infty }\alpha_{0,\mathrm{hk}}^{{{}^{\prime \prime }}^{2}}(t)\mathrm{dt} \end{array}$$

where is *l*(*β*_*hk*_, *γ*_*hk*_, *α*_0,*hk*_(·)) the full log likelihood, $\alpha_{0,\mathrm{hk}}^{\prime \prime }(t)$ is the second derivate of the baseline intensity function, and *κ*_*hk*_ is a positive smoothing parameter that controls the trade-off between the data fit and the smoothness of the functions. Maximization of (6) defines the maximum penalized likelihood estimators (MPnLEs). The estimator (MPnLEs) cannot be calculated explicitly but can be approximated on the basis of splines and the smoothing parameters *κ*_*hk*_ can be chosen by maximizing a likelihood cross-validation criterion as in Joly et al. [18]. The maximum penalized likelihood method is also implemented in the FrailtyPack R package [16].

##### Remark

In the presence of intensity functions with a joint frailty term in the model, a maximum penalized likelihood estimation is also used [11] and implemented in the FrailtyPack R package.

#### Model choice

Several models with different approaches have been defined: a *semi-Markovian* model (noted ( $P_{1}^{\zeta }$)) or a *non-homogeneous Markov model* (noted ( $P_{2}^{\zeta }$)) with a totally parametric approach or a semi-parametric approach. For example, we define $P_{1}^{\hat{\zeta }}$ and $P_{2}^{\hat{\zeta }}$ representing respectively the estimators of ( $P_{1}^{\zeta }$) and ( $P_{2}^{\zeta }$) based on a sample of i.i.d. observations $\left(O_{1},\ldots ,O_{n}\right)$ which come from the true unknown distribution *P*^∗^. To assess the risk of each estimator we propose to use the Expected Kullbak-Leibler risk (EKL) [6] defined as:

$$\text{EKL}\left(P_{1}^{\hat{\zeta }};O_{n+1}\right)=E_{P^{*}}\left[L_{O_{n+1}}^{P_{1}^{\hat{\zeta }}/P^{*}}\right]$$

where $L_{O_{n+1}}^{P_{1}^{\hat{\zeta }}/P^{*}}=\log{\frac{dP_{1}^{\hat{\zeta }}}{dP^{*}}}_{|O_{n+1}}$ is the log-likelihood ratio and $O_{n+1}$ is a new i.i.d observation. Commenges et al. [6] proposed the likelihood cross-validation (LCV) criterion to estimate this risk:

$$\mathrm{LCV}\left(P_{1}^{\hat{\zeta }}\right)=-\frac{1}{n}\overset{n}{\underset{i}{\sum }}\log{ℒ}_{O_{i}}^{P_{1}^{\hat{\zeta }-i}}$$

where ${ℒ}_{O_{i}}^{P_{1}^{\hat{\zeta }-i}}$ represents the likelihood of the observation $O_{i}$ based on the estimator $P_{1}^{\hat{\zeta }-i}$ defined without this observation.

Finally, to choose between the two estimators $P_{1}^{\hat{\zeta }}$ and $P_{2}^{\hat{\zeta }}$, we compute some differences of Expected Kullbak-Leibler risk (based on difference of LCV criterion). Commenges et al. [6] give an interpretation of the magnitude of these risks: values of 10^−1^, 10^−2^, 10^−3^, 10^−4^ are respectively qualified “large”, “moderate”, “small” and “negligible”. The FrailtyPack R package provides the LCV criterion for survival analysis (for us a transition *hk*). As we can estimate each transition separately, it is straightforward to get the LCV criterion for a particular Multi-state model. Concerning the selection of the covariates in each model, we use a manual selection procedure motivated by epidemiological/clinical knowledge and also based on statistical significance of hazard ratios.

#### Prediction

A posterior probability of any event can be easily derived from the multi-state model and its parameters. This probability, which can be computed for a new subject using a given set of covariates at the current time, and given a post-inclusion events, constitutes a dynamic tool of prediction. For instance, the aim may be to predict if VAP occurs between time *t*^∗^ and horizon time *t*^∗^ + *h*. The posterior probability of developing VAP on [*t*^∗^;*t*^*∗*^ + *h*] given the random effects $U_{i}=(u_{01}^{i},u_{02}^{i},u_{03}^{i})$ and the covariates $Z_{i}=(Z_{01}^{i},Z_{02}^{i},Z_{03}^{i})$ can be easily computed using the following expression with *k* = 1:

(7) $$\begin{array}{lll} \;\Pi_{0k}^{i}(t^{*},t^{*}+h|U_{i},Z_{i}) & =\text{Pr}(X(t^{*}+h)\;=\;1|X(t^{*})=0;U_{i},Z_{i})\; & \;\\ & =\int_{t^{*}}^{t^{*}+h}P_{00}(t^{*},t|U_{i},Z_{i})\; & \;\\ & \;\times \alpha_{0k}(t|u_{0k}^{i},Z_{0k}^{i})\mathrm{dt}\; \end{array}$$

with,

$$P_{00}\left(t^{*},t|U_{i},Z_{i}\right)=\text{exp}(-\int_{t^{*}}^{t}\overset{3}{\underset{k=1}{\sum }}\alpha_{0k}\left(v|u_{0k}^{i},Z_{0k}^{i}\right)\mathrm{dv})$$

The estimated posterior probabilities, ${\hat{\Pi }}_{0k}^{i}(t^{*},t^{*}+h|U_{i},Z_{i})\;i=1,\ldots ,N$ can be obtained by substituting the maximum penalized likelihood estimates of parameters *β*_0*k*_, *γ*_0*k*_, *α*_0,0*k*_, the empirical Bayes estimates for $u_{0k}^{i}$ and the individual information for the covariates $Z_{0k}^{i}$ by equation (7).

## Results

### Application revisited

The methodology exposed in the previous section is now applied to the OUTCOMEREA database. Table 1 gives first a description of the number of patients for each transition. The study initially included 2438 patients from 16 hospitals (between 3 and 1027 patients by centre). Patients were mostly discharged alive (69%), but there are also individuals who died in ICU (16%) or developed a nosocomial infection, a VAP (15%). The database contains many covariates that are related with the states modelled. Based on our previous studies [8,9,19,20] and literature reviews [21], we selected time-fixed covariates candidates as potential predictors of VAP, discharge and death in the ICU. A classical descending method was applied to select prognostic factors in each model (without frailties). This backward or descending elimination technique begins by calculating statistics for a model which includes all of the independent variables. Then the variables are deleted from the model one by one until all the variables remaining in the model produce statistics significant at the 0.10 level. At each step, the variable showing the smallest contribution to the model is deleted. In this application to assist in the variables interpretation, continuous covariates “age” and “Simplified Acute Physiology Score” (SAPS) were respectively transformed as categorial variables by median or quartiles. The different models were estimated using the FrailtyPack package developed by [16]. The Likelihood cross validation criterion is used to compare the different models. According to this criterion, the best is a semi-Markov model estimated by a semi-parametric approach (penalized likelihood estimator) with LCV = 4.044. Note that the difference of LCV between the semi-parametric estimator from the semi-Markov model and from the non-homogeneous Markov model (LCV = 4.055) is equal to 0.011 qualified as “moderate”. We have also compared a parametric approach versus a semi-parametric approach: for the non-homogenous model we obtained a difference of LCV equal to 0.407 qualified as “Large” between the parametric estimation (LCV = 4.462) and the semi-parametric estimation (LCV = 4.055); for the semi-Markov model we obtained a difference equal to 0.416 between the parametric estimation (LCV = 4.460) and the semi-parametric estimation (LCV = 4.044).

**Table 1:** Number of patients from the OUTCOMEREA database present in each transition

**Transition**	**No events**	**Events**	**(*%*)**	**Total**
01 (VAP)	2438	433	0.15	2871
02 (death without VAP)	2401	470	0.16	2871
03 (discharge without VAP)	903	1968	0.69	2871
12 (death with VAP)	314	119	0.27	433
13 (discharge with VAP)	119	314	0.73	433

Table 2 presents the semi-parametric estimation results of the semi-Markov model. A complete description of these selected covariates in this model is provided elsewhere ( [7]).

**Table 2:** **Result of the semi-markov model with penalized likelihood estimation incorporating frailty terms: HR (exp(*β*)) estimates and corresponding confidences intervals for the different transition intensities**

**Three independent frailties for transitions 01, 02 and 03**	**01 (VAP)**		**02 (death)**		**03 (discharge)**	
	**exp(*β*)**	**95%CI **	**exp(*β*)**	**95%CI**	**exp(*β*)**	**95%CI**
Sex (men=1)	1.51	(1.23-1.87)	-	-	0.85	(0.78-0.94)
*Age* ≥ 62	-	-	-	-	0.95	(0.86-1.05)
33 < *SAPSII* ≤ 45	-	-	1.62	(1.03-2.54)	0.66	(0.58-0.75)
45 < *SAPSII* ≤ 58	-	-	2.70	(1.75-4.14)	0.56	(0.49-0.64)
58 < *SAPSII*	-	-	4.83	(3.18-7.35)	0.40	(0.34-0.47)
Type of Admission :						
Elective surgery	1		-	-	1	
Emergency surgery	0.58	(0.41-0.83)	-	-	1.04	(0.90-1.20)
Medicine	0.89	(0.66-1.20)	-	-	0.96	(0.82-1.12)
Chronic diseases	-	-	1.37	(1.14-1.66)	0.84	(0.76-0.92)
Diabetes	1.48	(1.10-2.00)	1.30	(0.97-1.76)	-	-
*Diagnostic or symptoms on ICU admission:*						
ARDS	1.71	(1.16-2.54)	-	-	-	-
Trauma	2.52	(1.12-5.67)	-	-	-	-
Coma	1.23	(0.95-1.60)	2.90	(1.99-4.22)	1.06	(0.91-1.25)
Shock	1.21	(0.96-1.53)	2.12	(1.47-3.06)	0.73	(0.63-0.84)
Acute respiratory failure	-	-	1.79	(1.22-2.62)	0.65	(0.56-0.75)
*Use in the first 24 h of ICU admission :*						
Antimicrobials	0.61	(0.50-0.75)	0.66	(0.54-0.81)	0.86	(0.77-0.95)
Inotropes	-	-	-	-	0.74	(0.67-0.82)
Enteral nutrition	1.21	(0.97-1.50)	0.76	(0.60-0.95)	0.62	(0.55-0.71)
Parenteral nutrition	-	-	-	-	1.00	(0.87-1.14)
Variance of the frailty *γ* (SE)	0.19(0.11)		0.09(0.06)		0.15(0.07)	
**Two independent frailties for transitions 12 and 13**	**12 (death with VAP)**		**13 (discharge with VAP)**			
	**exp**(*β*)	**95%CI **	**exp**(*β*)	**95%CI**		
*Age* ≥ 62	-	-	0.84	(0.66-1.07)		
33 < *SAPSII* ≤ 45	2.13	(1.05-4.31)	-	-		
45 < *SAPSII* ≤ 58	2.60	(1.27-5.31)	-	-		
58 < *SAPSII*	4.81	(2.38-9.72)	-	-		
Parenteral nutrition	-		0.70	(0.51-0.97)		
Variance of the frailty *γ* (SE)	0.04(0.06)		0.11(0.08)			
**A joint subject-specific frailtyterm for transitions 12 and 13**	**12 (death with VAP)**		**13 (discharge with VAP)**			
	**exp(*β*)**	**95%CI **	**exp(*β*)**	**95%CI**		
*Age* ≥ 62	-	-	0.78	(0.61-0.98)		
33 < *SAPSII* ≤ 45	2.22	(0.99-4.97)	-	-		
45 < *SAPSII* ≤ 58	2.65	(1.17-6.01)	-	-		
58 < *SAPSII*	5.19	(2.31-11.65)	-	-		
Parenteral nutrition	-		0.67	(0.49-0.90)		
Common frailty variance *η* (SE)	0.77 (0.13)					
Power coefficient *ζ* (from *η*^*ζ*^)	-0.16 (0.29)					

−: the corresponding covariate has not been selected for this transition by the descendant strategy based on model without frailties. SAPS II = Simplified Acute Physiology Score, version II at admission (33, 45, and 58 separate the four quartiles); ARDS = Acute Respiratory Distress Syndrome; Acute Respiratory failure: indicate the need for respiratory supportive therapy; Coma: admission for a neurological disease and a Glasgow coma score of less than 9; Shock: admission in the ICU with sign or symptoms of shock according to common definitions.

We also fitted a semi-Markov model without taking into account the intra-centre correlation (results not shown). Using this model some factors were wrongly significantly associated. For instance, the effects of coma (Relative Risk= 1.39 (95%CI 1.07-1.79)), shock (Relative Risk= 1.28 (95%CI 1.01-1.62)) and the presence of an enteral nutritional therapy (Relative Risk= 1.24 (95%CI 1.00-1.53)) were incorrectly observed as significantly associated with the risk of VAP using the multi-state model without frailty term.

**Figure 2:**
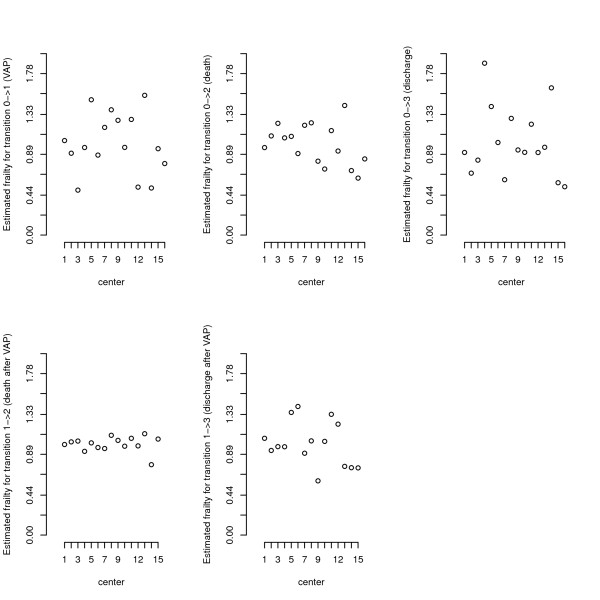
**Random centre-specific effect as estimated by posterior distribution for the different transitions.** The clusters have been ordered (ascending order) by the number of subjects in the cluster.

The variance of the frailty is significantly different from 0 for the transitions 01, 03 and not significantly different from 0 for the other transitions (Table 2). This means a significant heterogeneity of the transition rates (01, 03) between centres, but we did not observe a between-centre heterogeneity in the risk of death among patients with or without a VAP, nor for the risk of discharge with VAP. This is also depicted with the posterior frailty mean estimation for each centre in Figure 2. The number of subjects recruited greatly varied between centres (between 3 and 1027 patients by centre) therefore we also conducted an analysis excluding the two smallest hospitals (with 3 and 7 patients in each). The findings were very similar in terms of parameter’ estimates, and variance components.

We previously evaluated the heterogeneity between centres, but considering a different random effect for each transition of the multi-state model. We also fitted a joint frailty model for the two transitions 12 and 13, with a shared subject-specific random effect for the two transitions [11]. This approach allows us to simultaneously evaluate the prognostic effects of covariates on the two survival endpoints, discharge or death with a VAP. This joint frailty model accounts for the dependency between the two outcomes, and corrects for bias in the analysis due to dependency. Results are exposed at the bottom of Table 2. When comparing the joint model for transitions 12 and 13 to the reduced shared frailty models for the same transitions, covariates effects are similar, while the hazard ratio of SAPSII is slightly greater in the joint frailty model. This illustrates that ignoring the dependence between time to death and time to discharge may result in biases in the reduced shared frailty model compared to the joint model.

The variance of the joint random effect ( $\hat{\eta }=0.77$, one-sided Wald test = 0.77/0.13 = 5.92 > 1.64) is significantly different from 0 but the power coefficient *ζ* is not significantly different from 0 (two-sided Wald test  = 0.16/0.29 = 0.55 < 1.96). This shows that times of deaths and discharge are correlated, but with a slightly non-signicant negative association ( $\hat{\zeta }=-0.16$).

**Figure 3:**
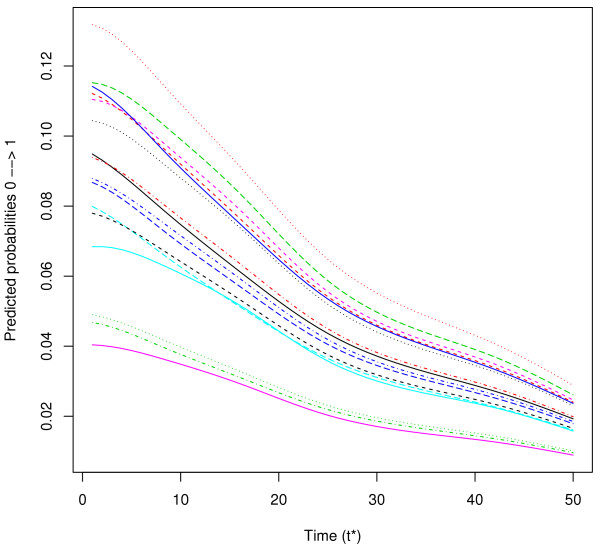
**Predicted probabilities (see formula (**7**)) of a patient in state 0 developing VAP between***t*∗**and***t*∗ + 3**days for a patient from the 16 different centers.** Predictions given for a men, aged > 62, SAPSII<33, with medicine admission, no chronic diseases, no diabetes, no ARDS, no Trauma, with shock, no acute respiratory failure, no antimicrobials, with inotropes, with enteral and parenteral nutrition.

To illustrate the use of posterior probabilities of VAP between times *t*∗ and *t*∗ + *h* given the information collected until time *t*∗, we show in Figure 3 the predicted probabilities for a patient in state 0 of developing VAP between times *t*∗ and *t*∗ + 3 days for patients of the same profile but belonging to each of the 16 centres. We observe a large difference of VAP probability according to the centre. These individual predictions underline the usefulness of dynamic prognostic tools that can take into account the clustering of observations.

### A Simulation study

#### Simulation details

In this section, we present a short simulation study, which in particular reveals the importance of taking into account the heterogeneity of the population for estimating the different intensity transition. We have considered the semi-Markov multi-state model (with four states) described in Figure 1. Each transition intensity is modelled by a proportional intensities model with a shared frailty term as defined in (4): the baseline intensity corresponds to the one of a Weibull distribution, the random effects follow a Gamma distribution and two covariates are used. These same two covariates are used for all the transitions: a binary variable modelled by a Binomial distribution with probability 0.5 (with specific effect $\beta_{\mathrm{hk}}^{1}$) and a continuous variable (with specific effect $\beta_{\mathrm{hk}}^{2}$) modelled by a Normal distribution (*μ* = 2 and *σ*^2^=0.1). The continuous variable is specific to the cluster; i.e. the same value for each subject belonging to the same cluster. There were either G=20, 30, 50 or 100 clusters with *n*_*i*_ = 75 or 150 subjects in each cluster. Two different values (0.15 and 0.30) of the variance of the frailty *γ*_*hk*_ are used for each transition. There were 500 simulated data sets for each case. The complete description of the simulation parameters is provided in Table 3.

**Table 3:** Simulation parameters of the semi-markov model

			**Transition**		
	**01**	**02**	**03**	**12**	**13**
*θ*_*hk*_=(*a*_*hk*_,*b*_*hk*_)	(1.3,15)	(1.3,35)	(1.25,15)	(1.3,45)	(1.25,41)
$\beta_{\mathrm{hk}}=(\beta_{\mathrm{hk}}^{1},\beta_{\mathrm{hk}}^{2})$	(0.8,1.0)	(0.6,1.2)	(1.3,0.3)	(0.7,1.1)	(0.6,1.2)

The parameter *θ*_*hk*_corresponds to the parameters of the Weibull distribution (shape parameter *a*_*hk*_and scale parameter *b*_*hk*_). The coefficient *β*_*hk*_is the parameter vector of the proportional hazard model for each transition *hk*.

Briefly, a simulation of a semi-Markov model consists of: (i) for each subject generate 3 times *T*_01_,*T*_02_ and *T*_03_ (representing respectively the occurrence of VAP, Death or Discharge) according to the intensities transitions defined in (4); (ii) if *T*_01_ ≠ min(*T*_01_,*T*_02_,*T*_03_) then the subject dies at time *T*_02_ (if *T*_02_ = min(*T*_01_,*T*_02_,*T*_03_)) or transits into the state Discharge (if *T*_03_ = min(*T*_01_,*T*_02_,*T*_03_)) and then back to (i) for a new subject; (iii) if *T*_01_ = min(*T*_01_,*T*_02_,*T*_03_) then the subject contracts VAP at time *T*_01_and then generates 2 new times (*T*_12_,*T*_13_) representing respectively the occurrence of Death or Discharge for a subject with VAP; (iv) if *T*_12_ = min(*T*_12_,*T*_13_) then the subject die at time *T*_01_ + *T*_12_ else the subject transits in the state Discharge at time *T*_01_ + *T*_13_; and then back to (i) for a new subject.

For simulation run, we estimated two parametric semi-markov models: 1) with specific frailty term in each transition and 2) without frailty term. For the two models, we computed the mean, the empirical standard errors (SEs), i.e. the SEs of estimates and the mean of the estimated SEs for ${\hat{\beta }}_{\mathrm{hk}}=({\hat{\beta }}_{\mathrm{hk}}^{1},{\hat{\beta }}_{\mathrm{hk}}^{2})$, and ${\hat{\gamma }}_{\mathrm{hk}}$.

#### Simulation results

The results of the simulation studies using parametric estimation are summarized in Table 4 and 5. To shorten the presentation of the results, we only present the case with G = 30 and 100 clusters. In general for the multi-state model incorporating frailty terms, the fixed effect $\beta_{\mathrm{hk}}=(\beta_{\mathrm{hk}}^{1},\beta_{\mathrm{hk}}^{2})$ and the variance *γ*_*hk*_of the frailties are well estimated. These bias decrease with the increasing sample size. The variability of estimates of *β*_*hk*_, measured by the empirical SEs or the mean of the estimated SEs, are similar. We observe the same result for the variability of estimates of *γ*_*hk*_. However, if we omit the frailties term in the estimation of the semi-Markov multi-state model, the estimators of the fixed effect *β*_*hk*_ are biased and the variability of estimates of *β*_*hk*_, measured by the mean of the estimated SEs, is lower than the variability measured by the empirical SEs. These results are clearly highlighted for the variable specific (representing by the effect *β*_2_) to the cluster (i.e. the same value for each subject belonging to the same cluster).

**Table 4:** **Estimates and standard errors (SE) according to the number of clusters*G*and the number of patients per cluster (*n***_***i***_**) for the parametric semi-Markov model integrating or not random effects (for M=500 simulated samples,*γ* = 0.15 and for simulation parameters explained in Table**3)

	**Mean**	**Mean**	**Mean**	**Empirical**	**Empirical**	**Empirical**	**Mean**	**Mean**	**Mean**
***h*→*k***	$\hat{\gamma }$	${\hat{\beta }}^{1}$	${\hat{\beta }}^{2}$	**SE(**$\hat{\gamma }$**S)**	**SE(**${\hat{\beta }}^{1}$**)**	**SE(**${\hat{\beta }}^{2}$**)**	**SE(**$\hat{\gamma }$**)**	**SE(**${\hat{\beta }}^{1}$**)**	**SE(**${\hat{\beta }}^{2}$**)**
*γ* = 0.15, *G* = 30*n*_*i*_ = 75									
*With frailties*									
01	0.139	0.798	1.002	0.042	0.064	0.081	0.042	0.062	0.082
02	0.134	0.599	1.204	0.052	0.089	0.093	0.049	0.089	0.091
03	0.133	1.300	0.291	0.055	0.100	0.103	0.052	0.098	0.096
12	0.141	0.697	1.094	0.055	0.100	0.094	0.053	0.096	0.099
13	0.138	0.597	1.200	0.049	0.083	0.098	0.047	0.080	0.092
*Without frailties*									
01		0.763	0.955		0.065	0.083		0.061	0.038
02		0.587	1.175		0.090	0.095		0.088	0.055
03		1.267	0.290		0.099	0.105		0.097	0.058
12		0.669	1.055		0.098	0.093		0.095	0.065
13		0.570	1.143		0.083	0.101		0.078	0.053
*γ* = 0.15, *G* = 30*n*_*i*_ = 150									
*With frailties*									
01	0.141	0.800	1.003	0.041	0.042	0.077	0.039	0.044	0.077
02	0.139	0.595	1.198	0.044	0.067	0.084	0.043	0.063	0.083
03	0.137	1.300	0.299	0.046	0.073	0.087	0.044	0.069	0.085
12	0.137	0.699	1.100	0.046	0.067	0.095	0.044	0.067	0.085
13	0.139	0.600	1.203	0.042	0.054	0.087	0.041	0.056	0.082
*Without frailties*									
01		0.766	0.961		0.043	0.078		0.043	0.027
02		0.584	1.168		0.068	0.086		0.063	0.039
03		1.267	0.296		0.072	0.094		0.068	0.041
12		0.673	1.064		0.069	0.097		0.066	0.045
13		0.572	1.146		0.055	0.091		0.055	0.037
*γ* = 0.15, *G* = 100*n*_*i*_ = 75									
*With frailties*									
01	0.147	0.801	1.001	0.023	0.035	0.044	0.024	0.034	0.044
02	0.149	0.598	1.201	0.026	0.051	0.052	0.029	0.049	0.050
03	0.146	1.299	0.293	0.031	0.052	0.054	0.031	0.053	0.052
12	0.146	0.697	1.099	0.031	0.054	0.056	0.030	0.053	0.053
13	0.147	0.602	1.202	0.029	0.042	0.050	0.027	0.044	0.049
*Without frailties*									
01		0.765	0.953		0.036	0.044		0.034	0.020
02		0.585	1.168		0.051	0.052		0.048	0.029
03		1.262	0.291		0.054	0.056		0.053	0.030
12		0.670	1.059		0.053	0.055		0.052	0.034
13		0.573	1.139		0.044	0.052		0.043	0.028
*γ*=0.15, *G*=100*n*_*i*_=150									
*With frailties*									
01	0.148	0.800	0.997	0.022	0.024	0.041	0.022	0.024	0.042
02	0.147	0.601	1.200	0.025	0.036	0.047	0.024	0.034	0.045
03	0.146	1.302	0.297	0.025	0.039	0.045	0.026	0.038	0.046
12	0.148	0.702	1.103	0.025	0.037	0.049	0.025	0.037	0.047
13	0.147	0.602	1.204	0.024	0.031	0.045	0.024	0.031	0.045
*Without frailties*									
01		0.763	0.949		0.025	0.041		0.024	0.014
02		0.588	1.168		0.036	0.049		0.034	0.021
03		1.265	0.295		0.039	0.051		0.037	0.021
12		0.674	1.063		0.037	0.049		0.036	0.024
13		0.573	1.139		0.032	0.048		0.030	0.020

**Table 5:** **Estimates and standard errors (SE) according to the number of clusters*G*and the number of patients per cluster (*n***_***i***_**) for the parametric semi-Markov model integrating or not random effects (for M=500 simulated samples,*γ*=0.30 and for simulation parameters explained in Table**3**)**

	**Mean**	**Mean**	**Mean**	**Empirical**	**Empirical**	**Empirical**	**Mean**	**Mean**	**Mean**
***h*→*k***	$\hat{\gamma }$	${\hat{\beta }}^{1}$	${\hat{\beta }}^{2}$	**SE(**$\hat{\gamma }$**)**	**SE(**${\hat{\beta }}^{1}$**)**	**SE(**${\hat{\beta }}^{2}$**)**	**SE(**$\hat{\gamma }$**) **	**SE(**${\hat{\beta }}^{1}$**)**	**SE(**${\hat{\beta }}^{2}$**)**
*γ* = 0.30, *G* = 30*n*_*i*_ = 75									
*With frailties*									
01	0.281	0.800	1.004	0.079	0.064	0.114	0.076	0.063	0.109
02	0.285	0.611	1.201	0.087	0.091	0.125	0.087	0.089	0.118
03	0.276	1.302	0.296	0.093	0.096	0.126	0.090	0.098	0.121
12	0.276	0.697	1.106	0.093	0.098	0.128	0.088	0.097	0.123
13	0.277	0.599	1.207	0.084	0.082	0.117	0.082	0.081	0.117
*Without frailties*									
01		0.734	0.917		0.068	0.114		0.061	0.037
02		0.586	1.141		0.092	0.128		0.088	0.053
03		1.235	0.297		0.097	0.135		0.096	0.057
12		0.646	1.032		0.101	0.133		0.094	0.063
13		0.547	1.096		0.086	0.126		0.079	0.052
*γ* = 0.30, *G* = 30*n*_*i*_ = 150									
*With frailties*									
01	0.281	0.803	0.999	0.077	0.044	0.107	0.073	0.044	0.106
02	0.278	0.599	1.197	0.078	0.065	0.116	0.077	0.062	0.109
03	0.280	1.304	0.300	0.082	0.073	0.124	0.080	0.069	0.112
12	0.274	0.699	1.103	0.078	0.069	0.115	0.078	0.068	0.112
13	0.285	0.601	1.200	0.080	0.056	0.116	0.077	0.057	0.111
*Without frailties*									
01		0.737	0.912		0.051	0.107		0.044	0.026
02		0.576	1.138		0.067	0.121		0.062	0.038
03		1.236	0.291		0.078	0.139		0.068	0.040
12		0.648	1.027		0.071	0.123		0.066	0.045
13		0.546	1.090		0.062	0.127		0.056	0.037
*γ* = 0.30, *G* = 100*n*_*i*_ = x75									
*With frailties*									
01	0.293	0.800	1.003	0.045	0.034	0.060	0.043	0.034	0.059
02	0.294	0.596	1.196	0.047	0.047	0.060	0.049	0.049	0.063
03	0.296	1.304	0.308	0.053	0.054	0.067	0.051	0.053	0.066
12	0.293	0.699	1.100	0.050	0.052	0.067	0.050	0.053	0.066
13	0.293	0.600	1.200	0.049	0.046	0.063	0.047	0.044	0.064
*Without frailties*									
01		0.732	0.912		0.037	0.062		0.034	0.020
02		0.570	1.130		0.049	0.065		0.048	0.028
03		1.230	0.305		0.056	0.074		0.052	0.030
12		0.644	1.017		0.055	0.073		0.051	0.033
13		0.544	1.081		0.048	0.068		0.043	0.028
*γ*=0.30, *G*=100*n*_*i*_=150									
*With frailties*									
01	0.291	0.799	1.004	0.041	0.023	0.057	0.041	0.024	0.057
02	0.292	0.600	1.201	0.043	0.036	0.063	0.044	0.034	0.060
03	0.292	1.301	0.302	0.046	0.037	0.065	0.045	0.037	0.061
12	0.294	0.697	1.099	0.044	0.037	0.063	0.045	0.037	0.062
13	0.294	0.601	1.202	0.043	0.030	0.058	0.043	0.031	0.060
*Without frailties*									
01		0.733	0.913		0.028	0.059		0.024	0.014
02		0.574	1.135		0.037	0.068		0.034	0.020
03		1.230	0.298		0.039	0.071		0.037	0.021
12		0.642	1.017		0.038	0.068		0.036	0.024
13		0.543	1.079		0.032	0.067		0.031	0.020

## Discussion

We have described a multistate model with frailty terms to account for heterogeneity between clusters on each transition. Such models appear promising in the setting of competing risk analyses using clustered data (i.e., multi-centre clinical trials, meta-analysis). Lack of software is a potential obstacle. We propose here a tractable model, semi-Markov as well as non-homogenous Markov, with semi-parametric or parametric estimates. The model can be readily derived with the R package FrailtyPack a simple and free approach, which does not require any time-consuming calculation. We also proposed a measure of models selection which evaluates the relative goodness of fit among a collection of models. To give an example, we provided a R code for simulating a data set and to analyze it (see Additional file 1).

Vital status and time of death or time of discharge in ICU are known exactly. However, there may be more complex schemes with interval-censored times to events, i.e., the event occurs in a known time interval *L,R*. The semi-Markov or non-homogenous Markov models discussed in this paper do not allow the direct treatment of these interval-censored data. It would be interesting to extend the multistate frailty model in the case of interval-censoring [22]. This would lead to numerical integration in the estimation process due to the lack of a closed-form solution of the multiple integrals, and this can be very time-consuming especially when the number of states rises. Also for large numbers of states, it is clear that substantial datasets are required for frailty variance estimation.

The proposed approach can also be used to predict probabilities of future events, given a patient’s history, covariates, and random effets, using parameter estimates and the estimates of corresponding baseline hazards and survival functions. Open research questions include prediction assessment with time-dependent prognostic factors. The aim would be to develop an updating mechanism which would allow dynamic updating of the predictions for a given patient in case of important changes in biomarkers.

A recent article discussed the identifiability and the (im)possibilities of frailties in multi-state models [23] but without considering covariates. They also compared predictive accuracy of different multi-state models with or without frailties using a k-fold cross validated partial log-likelihood [24]. They obtained that frailty models showed the best predictive performance in the comparison.

VAP represents an important and challenging example in which multistate frailty models should be used. This nosocomial infection is very frequent in ICU and is associated with an increase in ICU mortality, length of stay and cost. Many risk factors have been described in the past, and some new preventive interventions have been tested with often conflicting results [25]. This result could be due to the absence of control of appropriate confounding factors, to the heterogeneity of effects according to ICU subpopulations or to discrepancies in the diagnostic procedure. Indeed the diagnosis is based on the physician’s behaviour when clinical, biological and radiological signs of pneumonia occur. Even when diagnostic criteria are fixed in randomized control trials, the reported incidences vary from nine to 70%. Even if all fixed covariates are taken into account, and definitions carefully followed, the incidence densities still vary from 9.7 to 26.1 per 1000 mechanical-ventilation days [26]. The use of frailty terms in multistate models might then be important to unmask residual sources of variability (centre effect) when looking for risk factors of VAP. It may also be useful to compare incidences of VAP between hospitals if used as a quality indicator. It may also be important to explain the huge variability in the estimation of the attributable mortality of the disease.

Since the multi-state model under consideration contains cluster-specific random effects, the definition of the predictions is not straightforward. The proposed posterior prediction probabilities may be used to predict survival functions of subjects from existing clusters. In this cluster focus, the random effects $u_{\mathrm{hk}}^{i}$ are themselves of interest, and they are parameters to be estimated. In contrast, when the interest is on predicting survival for patients from new clusters, a marginal approach is better and corresponds to a population focus rather than a cluster focus. In this population focus, each conditional survival function is replaced by its marginal version. The marginal or observable survival function for the shared gamma frailty model (4) is $S(t|Z_{\mathrm{hk}}^{\mathrm{ij}})=E_{u}[S(t|Z_{\mathrm{hk}}^{\mathrm{ij}},u_{\mathrm{hk}}^{i})]=$$\frac{1}{{\left(1+\gamma_{\mathrm{hk}}\Lambda_{\mathrm{hk}}^{\mathrm{ij}}\left(t\right)\right)}^{1/\gamma_{\mathrm{hk}}}}$ with $\Lambda_{\mathrm{hk}}^{\mathrm{ij}}(t)$ the cumulative transition intensity function specific to the transition *hk*.

## Conclusions

The use of multistate frailty models allows the simple analysis of very complex data. Such models could help improve the estimation of the impact of proposed prognostic features on each transition in a multi-centre study. We have suggested a method and software that gives accurate estimates and enables inference for any parameter or predictive quantity of interest.

## Competing interests

The authors declare that they have no competing interests.

## Authors’ contributions

BL and VR developed the methodology, performed the simulation and the analysis on the dataset as well as wrote the manuscript. JFT collected and interpreted the dataset as well as wrote the description and the interpretation on the Application section. All authors read and approved the final manuscript.

## Pre-publication history

The pre-publication history for this paper can be accessed here:

http://www.biomedcentral.com/1471-2288/12/79/prepub

## Supplementary Material

Additional file 1 We provide a R code for simulating a data set and to analyze it: file name “supplementary-material-R-code.R”. Click here for file
